# Minichromosome Maintenance Protein 7 is a potential therapeutic target in human cancer and a novel prognostic marker of non-small cell lung cancer

**DOI:** 10.1186/1476-4598-10-65

**Published:** 2011-05-28

**Authors:** Gouji Toyokawa, Ken Masuda, Yataro Daigo, Hyun-Soo Cho, Masanori Yoshimatsu, Masashi Takawa, Shinya Hayami, Kazuhiro Maejima, Makoto Chino, Helen I Field, David E Neal, Eiju Tsuchiya, Bruce AJ Ponder, Yoshihiko Maehara, Yusuke Nakamura, Ryuji Hamamoto

**Affiliations:** 1 Laboratory of Molecular Medicine, Human Genome Center, Institute of Medical Science, The University of Tokyo, 4-6-1 Shirokanedai, Minato-ku, Tokyo 108-8639, Japan; 2 Department of Surgery and Science, Graduate School of Medical Science, Kyusyu University, 3-1-1 Maidashi, Higashi-ku, Fukuoka 812-8582, Japan; 3 Department of Medical Oncology, Shiga University of Medical Science, Otsu 520-2192, Japan; 4 Specialty Chemicals & International Division Pharmaceuticals Group, Nippon Kayaku Co., Ltd., 11-2, Fujimi 1 Chome, Chiyoda-ku, Tokyo, 102-8172, Japan; 5 Department of Genetics, University of Cambridge, Downing Street, Cambridge CB2 3EH, UK; 6 Department of Oncology, Cancer Research UK Cambridge Research Institute, University of Cambridge, Robinson Way, Cambridge CB2 0RE, UK; 7 Department of Pathology, Saitama Cancer Center, Saitama 362-0806, Japan; 8 Molecular Pathology and Genetics Division, Kanagawa Cancer Center Research Institute, Kanagawa 241-0815, Japan

## Abstract

**Background:**

The research emphasis in anti-cancer drug discovery has always been to search for a drug with the greatest antitumor potential but fewest side effects. This can only be achieved if the drug used is against a specific target located in the tumor cells. In this study, we evaluated Minichromosome Maintenance Protein 7 (MCM7) as a novel therapeutic target in cancer.

**Results:**

Immunohistochemical analysis showed that MCM7 was positively stained in 196 of 331 non-small cell lung cancer (NSCLC), 21 of 29 bladder tumor and 25 of 70 liver tumor cases whereas no significant staining was observed in various normal tissues. We also found an elevated expression of MCM7 to be associated with poor prognosis for patients with NSCLC (*P *= 0.0055). qRT-PCR revealed a higher expression of *MCM7 *in clinical bladder cancer tissues than in corresponding non-neoplastic tissues (*P *< 0.0001), and we confirmed that a wide range of cancers also overexpressed *MCM7 *by cDNA microarray analysis. Suppression of MCM7 using specific siRNAs inhibited incorporation of BrdU in lung and bladder cancer cells overexpressing MCM7, and suppressed the growth of those cells more efficiently than that of normal cell strains expressing lower levels of MCM7.

**Conclusions:**

Since MCM7 expression was generally low in a number of normal tissues we examined, MCM7 has the characteristics of an ideal candidate for molecular targeted cancer therapy in various tumors and also as a good prognostic biomarker for NSCLC patients.

## Background

The emergence of effective cancer chemotherapy is one of the major medical advances of late years [1]. Adjuvant chemotherapy for lung, breast or colon cancer can augment the survival benefit afforded by surgical management [2-4]. Even in patients with advanced solid tumors or recurrences following surgery, chemotherapy can offer lengthened survival of worthwhile quality. In those patients, however, the therapeutic index is narrow: responses are usually partial, often disappointingly brief and unpredictable [5]. In addition, many antitumor agents have also been troubled with issues of sometimes unexpected side effects. These circumstances accentuate the limitation of cytotoxic chemotherapy. Therefore, it remains essential to discover novel therapeutic targets to extend the capability of cancer chemotherapy.

DNA replication in eukaryotic cells is a highly regulated process that ensures the accurate duplication of genetic information while preserving genome stability. A large number of molecular players, including minichromosome maintenance (MCM) proteins, are involved in DNA replication [6-8]. MCM proteins are essential replication initiation and elongation factors originally found in *Saccharomyces cerevisiae*, existing in a functional complex consisting of MCM2-7. They are evolutionarily conserved in all eukaryotes [9]. The MCM complex belongs to the AAA+ (ATPases associated with various cellular activities) family, and ensures that DNA undergoes a single round of replication per cell cycle by a licensing mechanism [9-13]. The MCM4, 6 and 7 subcomplex possesses DNA helicase activity that promotes unwinding of double stranded DNA at the replication forks [14-16]. Although it is known that aberrant DNA replication leads to pathological disorders including cancer, how dysregulated MCM proteins contribute to carcinogenesis, and aggressive cancer with poor prognosis, is unclear.

In this study, we demonstrate that dysregulation of MCM7 expression is observed in various types of cancer and correlates with a negative outcome in patients with NSCLC after surgical resection. This work forms the basis for further functional studies, which will explore MCM7 as a potential therapeutic and prognostic target in lung and other cancers.

## Materials and methods

### Cell lines and lung tissue samples

Cancer cell lines used in this study were as follows: lung adenocarcinoma (ADC) NCI-H1781, NCI-H1373, LC319, A549 and PC-14; lung squamous cell carcinoma (SCC) SK-MES-1, NCI-H2170, NCI-H520, NCI-H1703 and RERF-LC-AI; lung large cell carcinoma (LCC) LX1; small cell lung cancer (SCLC) SBC-3, SBC-5, DMS273 and DMS114; bladder cancer SW780, RT4 and 5637; liver cancer HepG2, SNU475 and Huh7; colorectal cancer SW480, LoVo and HCT116. Human small airway epithelial cells (SAECs), normal human lung fibroblasts (IMR-90, WI-39 and HFL-1) and normal human colon fibroblasts (CCD-18Co) were used as normal control cells. All cell lines were grown in monolayers in appropriate media supplemented with 10% fetal bovine serum and 1% antibiotic/antimycotic solution (Sigma). All cell lines were maintained at 37°C in humid air with 5% CO_2 _(except for SW780), or without CO_2 _(SW780). Cells were transfected with FuGENE6™ (Roche Applied Science, Penzberg, Germany) according to the manufacturer's protocols. Primary non-SCLC (NSCLC) tissue samples as well as their corresponding normal tissues adjacent to resection margins, from patients having no anticancer treatment before tumor resection, had been obtained earlier with informed consent [17-19]. All tumors were staged on the basis of the pathologic tumor-node-metastasis classification of the International Union Against Cancer. A total of nine frozen primary lung cancer tissues for RNA extraction were obtained as published earlier [18,19]. Formalin-fixed primary lung tumors and adjacent normal lung tissue samples used for immunostaining on tissue microarrays had been obtained from 331 patients undergoing curative surgery at Saitama Cancer Center (Saitama, Japan) [20,21]. To be eligible for this study, tumor samples were selected from patients who fulfilled all of the following criteria: (a) patients suffered primary NSCLC with histologically confirmed stage (only pT1 to pT4, pN0 to pN2, and pM0); (b) patients underwent curative surgery, but did not receive any preoperative treatment; (c) among them, NSCLC patients with positive lymph node metastasis (pN1-pN3) were treated with platinum-based adjuvant chemotherapies after surgical resection, whereas patients with pN0 did not receive adjuvant chemotherapies; and (d) patients whose clinical follow-up data were available. This study and the use of all clinical materials mentioned were approved by individual institutional ethics committees.

### Bladder tissue samples and RNA preparation

Bladder tissue samples and RNA preparation were described previously [22-24]. Briefly, 136 surgical specimens of primary urothelial carcinoma were collected, either at cystectomy or transurethral resection of bladder tumor (TURBT), and snap frozen in liquid nitrogen. Twenty-three specimens of normal bladder urothelial tissue were collected from areas of macroscopically normal bladder urothelium in patients with no evidence of malignancy. Approximately 10,000 cells were microdissected from both stromal and epithelial/tumor compartments in each tissue. RNA was extracted using an RNeasy Micro Kit (QIAGEN, Crawley, UK). Areas of cancer or stroma containing significant inflammatory areas of tumor or stroma containing significant inflammatory cell infiltration were avoided to prevent contamination [24]. Use of tissues for this study was approved by Cambridgeshire Local Research Ethics Committee (Ref 03/018).

### Expression profiling in cancer using cDNA microarrays

We established a genome-wide cDNA microarray with 36,864 cDNAs selected from UniGene database of the National Center for Biotechnology Information (NCBI). This microarray system was constructed essentially as described previously [18,25,26]. Briefly, the cDNAs were amplified by RT-PCR using poly (A)^+ ^RNAs isolated from various human organs as templates; the lengths of the amplicons ranged from 200 to 1,100 bp, without any repetitive or poly (A) sequences. Many types of tumor and corresponding non-neoplastic tissues were prepared in 8-μm, as described previously [25]. A total of 30,000 - 40,000 cancer or noncancerous cells were collected selectively using the EZ cut system (SL Microtest GmbH, Jena, Germany) according to the manufacturer's protocol. Extraction of total RNA, T7-based amplification, and labeling of probes were performed as described previously [25]. A measure of 2.5-μg aliquots of twice-amplified RNA (aRNA) from each cancerous and noncancerous tissue was then labeled, respectively, with Cy3-dCTP or Cy5-dCTP.

### Quantitative real-time PCR

As described previously, we prepared 136 bladder cancer and 23 normal bladder tissues in Addenbrooke's Hospital, Cambridge [27]. Specific primers for human *GAPDH *(housekeeping gene) and *MCM7 *were designed (primer sequences in Additional file 1). PCR reactions were performed using the LightCycler^® ^480 System (Roche Applied Science) following the manufacture's protocol.

### siRNA transfection and cell growth assay

siRNA oligonucleotide duplexes were purchased from Sigma-Aldrich (St. Louis, MO, USA) for targeting the human *MCM7 *transcripts. siEGFP and siNegative control (siNC), which consists of three different oligonucleotide duplexes, were used as control siRNAs. The siRNA sequences are described in Additional file 2. siRNA duplexes (100 nM final concentration) were transfected into lung and bladder cancer cell lines with Lipofectamine 2000 (Life Technologies, Carlsbad, CA, USA) for 72 h, and cell growth was examined using the Cell Counting Kit-8 (Dojindo, Kumamoto, Japan) [28].

### Western blot analysis

Whole cell lysates were prepared from the cells with RIPA-like buffer, and total protein (10 μg) was transferred to nitrocellulose membrane. The membrane was probed with anti-MCM7 antibody (141.2, Santa Cruz Biotechnology, Santa Cruz, CA, USA). ACTB (I-19, Santa Cruz Biotechnology) was used to ensure equal loading and transfer of proteins. Protein bands were detected by incubating with horseradish peroxidase-conjugated antibodies (GE Healthcare, Little Chalfont, UK) and visualizing with Enhanced Chemiluminescence (GE Healthcare).

### BrdU labeling and immuocytochemical analysis

BrdU labeling and immunocytochemistry were performed according to previously reported protocols [23,27,29]. A549, SBC5 and SW780 cells were incubated with appropriate media containing 2 μM BrdU (BD Biosciences, Franklin Lakes, NJ, USA) for 20 min, and fixed and permeabilized with 100% methanol for 5 min at room temperature. The cells were washed with PBS, and then blocked by 3% BSA for 1 h at 37°C. Then, the cells were incubated with an anti-MCM7 antibody in 3% BSA overnight at 4°C. After incubation with 1^st ^antibody, the cells were reacted with Alexa Fluor 594-conjugated goat anti-mouse IgG for 1 h at 37°C in the blocking solution. They were then re-fixed, treated with 4 M HCl for 30 min at room temperature and incubated with FITC-conjugated anti-BrdU (BD Biosciences), diluted 1:300, for 1 h at room temperature, followed by observation with confocal microscopy (Leica Microsystems, Wetzlar, Germany).

### Immunohistochemical staining and tissue microarray

Immunohistochemical analysis was performed using a specific mouse-MCM7 antibody as described previously [27,30]. For clinical lung cancer tissues and liver tissue microarray (70 cases), ENVISION+ kit/horseradish peroxidase (Dako, Glostrup, Denmark) was applied. VECTASTAIN^® ^ABC KIT (Vector Laboratories, Burlingame, CA, USA) was used for bladder tissue microarray and normal tissue slides. Tumor tissue microarrays were constructed with 331 primary NSCLCs which had been obtained by a single institutional group (see above) with an identical protocol to collect, fix, and preserve the tissues after resection [31-33]. Considering the histologic heterogeneity of individual tumors, a tissue area for sampling was selected based on visual alignment with the corresponding H&E-stained section on a slide. Three, four or five tissue cores (diameter, 0.6 mm; depth, 3-4 mm) taken from a donor tumor block were placed into a recipient paraffin block with a tissue microarrayer (Beecher Instruments, Sun Prairie, WI, USA). A core of normal tissue was punched from each case, and 5-μm sections of the resulting microarray block were used for immunohistochemical analysis. Three independent investigators semiquantitatively assessed MCM7 positivity without prior knowledge of clinicopathologic data. Because the intensity of staining within each tumor tissue core was mostly homogeneous, the intensity of MCM7 staining was semiquantitatively evaluated using the following criteria: negative (no appreciable staining in tumor cells) and positive (brown staining appreciable in more than 30% of the nucleus of tumor cells). Cases were accepted as positive only if all reviewers independently defined them as such.

### Statistical analysis

The Kruskal-Wallis test was used to examine the difference between several independent subgroups. Student's *t*-test or Mann-Whitney's *U*-test was used to analyze the difference between two independent subgroups. Survival curves were calculated from the date of surgery to the time of death related to NSCLC or to the last follow-up observation. Kaplan-Meier curves were calculated for each relevant variable and for MCM7 expression; differences in survival times among patient subgroups were analyzed using the log-rank test. Univariate and multivariate analyses were done with the Cox proportional hazard regression model to determine associations between clinicopathological variables and cancer-related mortality. First, we analyzed associations between death and possible prognostic factors including age, gender, histology, pT classification and pN classification, taking into consideration one factor at a time. Second, multivariate Cox analysis was applied on backward (stepwise) procedures that always forced strong MCM7 expression into the model, along with any and all variables that satisfied an entry level of *P *< 0.05. As the model continued to add factors, independent factors did not exceed an exit level of *P *< 0.05.

## Results

### MCM7 expression is significantly high in lung cancer tissues and correlated with poor prognosis in NSCLC

**Figure 1:**
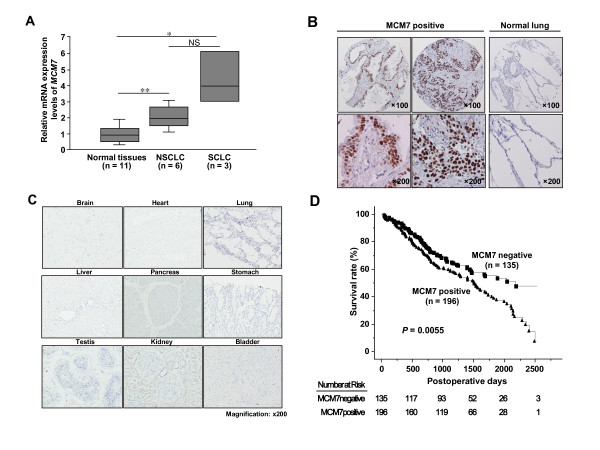
**Dysregulation of MCM7 in lung cancer tissues, and correlation of MCM7 expression to a negative outcome for NSCLC patients after surgical operation**. (A) Overexpression of *MCM7 *in clinical lung cancer tissues [NSCLC (n = 6) and SCLC (n = 3)] compared with several normal tissues (lung, brain, colon, esophagus, eye, heart, liver, rectum, stomach, bladder and kidney) analyzed by quantitative real-time PCR. Statistical analysis was done using Kruskal-Wallis test; *P *< 0.0001: Mann Whitney *U*-test; *, *P *< 0.05; NS, not significant (*P *= 0.0528). (B) Representative cases for positive MCM7 expression in lung ADC, SCC tissues and normal lung tissues. Original magnification, ×100 and ×200. (C) Immunohistochemical analysis of MCM7 in various normal tissues. No significant staining was observed. Characteristics of each tissue were described in Additional file 12. (D) Kaplan-Meier estimates of overall survival time of patients with NSCLC (*P *= 0.0055, log-rank test).

We previously reported that PRMT6, a type I arginine methytransferase, is involved in human carcinogenesis [27]. To clarify detailed functions of PRMT6 in cancer, we performed IP-MS analysis and identified MCM7 as a binding partner (data not shown). The interaction was confirmed by co-immunoprecipitation and immunocytochemistry (Additional file 3). Intriguingly, quantitative real-time PCR showed that expression levels of *MCM7 *in 9 lung cancer tissues (6 NSCLC cases and 3 SCLC cases) were significantly higher than those in 11 normal tissues containing lung, brain, colon, esophagus, eye, liver, rectum, stomach, bladder and kidney (Figure 1A). Therefore, we hypothesized that MCM7 can also be involved in human carcinogenesis. To validate these results, we conducted immunohistochemical analysis on tissue microarray containing tissue sections from 331 NSCLC patients, who had undergone surgical resection. Immunohistochemistry using an MCM7-specific antibody showed nuclear localization in cancer tissues, but nothing was detected in normal lung tissues (Figure 1B). Importantly, specific MCM7 signals were hardly detected in normal brain, heart, lung, liver, pancreas, stomach, testis, kidney and bladder tissues (Figure 1C), indicating that MCM7 may be specifically overexpressed in cancer tissues. Of 331 cases, MCM7 stained positively in 196 cases (61.1%) and negatively in 135 cases (38.9%; Table 1). Subsequently, we analyzed the association of MCM7 expression with clinical outcomes, and found that expression of MCM7 in NSCLC patients was significantly associated with male gender (*P *< 0.0001, Fisher's exact test; Table 1), non-adenocarcinoma (ADC) histology (*P *< 0.0001), presence of lymph node metastasis (pN1-2; *P *< 0.0001) and tumor-specific 5-year survival after the resection of primary tumors (*P *= 0.0055 by log-rank test; Figure 1D). We then applied univariate analysis to evaluate associations between patient prognosis and several factors, including MCM7 expression, age, gender, histologic type (non-ADC versus ADC), pT stage (tumor size, T1 versus T2 + T3) and pN stage (node status, N0 versus N1+N2). All those parameters were significantly associated with poor prognosis (Table 2). However, multivariate analysis revealed that MCM7 status did not show the statistical significance as an independent prognostic factor for surgically treated NSCLC patients enrolled in this study, whereas age, pT and pN stages did so (Table 2). This result might be due to the preponderance of MCM7 expression up to the pN stage.

**Table 1:** Association between MCM7-positive in NSCLC and characteristics of patients

		Total	MCM7 positive	MCM7 negative	*P*-value* positive vs negative
		**n = 331**	**n = 196**	**n = 135**	

Gender					
	Male	236	156	80	<0.0001
	Female	95	40	55	

Age(years)					
	<65	168	107	61	NS
	≧65	163	89	74	

Histological type					
	ADC	214	101	113	
	SCC	78	63	15	<0.0001^†^
	Others**	39	32	7	

pT factor					
	T1T2	227	127	100	NS
	T3T4	104	69	35	

pN factor					
	N0	197	99	98	<0.0001
	N1+N2	134	97	37	

Abbreviations: ADC, adenocarcinoma; SCC, squamous-cell carcinoma; NS, not siginificant.

*Fisher's exact test.

**Others: large-cell carcinoma (LCC) and adenosquamous-cell carcinoma (ASC).

^†^ADC vs non-ADC.

**Table 2:** Cox's proportional hazards model analysis of prognostic factors in patients with NSCLC

	Variables	Hazards ratio	95%Cl	Unfavorable/Favorable	*P*-value*
Univariate analysis					
	MCM7	1.546	1.134-2.110	Positive/Negative	0.0059
	Age (years)	1.354	1.008-1.818	65 ≧/< 65	0.0442
	Gender	1.435	1.020-2.020	Female/Male	0.0378
	Histological type	1.530	1.138-2.056	nonADC/ADC	0.0048
	pT factor	1.647	1.217-2.232	T3T4/T1T2	0.0013
	pN factor	2.617	1.945-3.521	N1+N2/N0	<0.0001

	**Variables**	**Hazards ratio**	**95%Cl**	**Unfavorable/Favorable**	*P*-value*

Multivariate analysis					
	MCM7	0.935	0.665-1.314	Positive/Negative	NS
	Age (years)	1.576	1.166-2.130	65 ≧/< 65	0.0031
	Gender	0.801	0.553-1.159	Female/Male	NS
	Histological type	0.819	0.589-1.140	nonADC/ADC	NS
	pT factor	1.538	1.131-2.092	T3T4/T1T2	0.006
	pN factor	2.577	1.887-3.521	N1+N2/N0	<0.0001

Abbreviations: ADC, adenocarcinoma; NS, not siginificant.

*Fisher's exact test.

### MCM7 is overexpressed in bladder and various types of cancers

**Figure 2:**
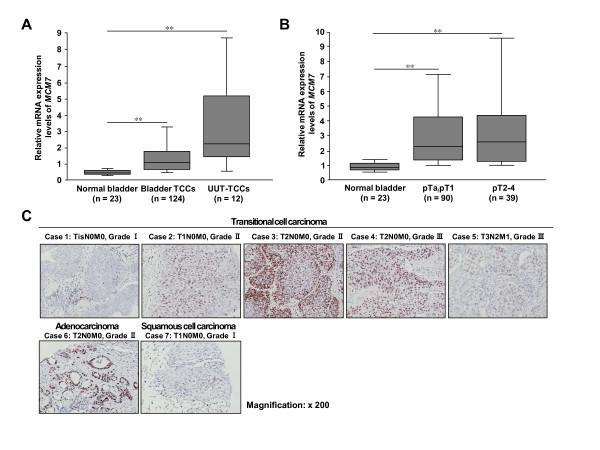
**Elevated MCM7 expression in bladder cancer**. (A) mRNA expression levels of *MCM7 *in 124 bladder transitional cell carcinomas (TCCs), 12 upper urinary tract transitional cell carcinomas (UUT-TCCs) and 23 normal bladder tissues analyzed by quantitative real-time PCR. Statistical analysis was done using Kruskal-Wallis test; *P *< 0.0001: Mann Whitney *U-*test; **, *P *< 0.0001. (B) Correlation between *MCM7 *expression and pathological tumor stages. Statistical analysis using Kruskal-Wallis test; *P *< 0.0001: Mann Whitney *U-*test; **, *P *< 0.0001. (C) Immunohistochemical analysis of MCM7 in bladder cancer tissues. Original magnification, ×200.

In addition to lung tissues, we examined expression levels of MCM7 in bladder tissues. Quantitative real-time PCR analysis using 23 normal bladder tissues, 124 bladder transitional cell carcinomas (TCCs) and 12 upper urinary tract transitional cell carcinomas (UUT-TCCs) showed elevated mRNA levels of *MCM7 *in bladder and especially UUT-TCCs compared with normal bladder tissues (Figure 2A). Although there were significant differences between normal bladder and bladder cancers of all pT stages, no significant differences were observed between each pT stage nor tumor grade (Figure 2B; data not shown). Subsequently, immunohistochemical analysis using a bladder tissue microarray revealed that MCM7 was up-regulated in bladder cancer tissues at the protein level (21/29, 72.4%; Figure 2C and Additional file 4). In our microarray expression analysis, we confirmed elevated MCM7 expression in various types of cancers including lung, bladder and liver (Table 3), and tissue microarray immunohistochemical analysis showed up-regulation of MCM7 in liver cancer tissues at the protein level (25/70, 35.7%; Additional files 5 and 6).

**Table 3:** Gene expression profile of *MCM7 *in cancer tissues analyzed by cDNA microarray*

		Ratio (Tumor/Normal)		
		
Tissue type	Case (n)	Count > 2 (T/N)	Count > 3 (T/N)	Count > 5 (T/N)
NSCLC	37	9 (24.3%)	4 (10.8%)	0 (0%)
SCLC	15	12 (80.0%)	9 (60.0%)	2 (13.3%)
Esophageal cancer	62	37 (59.7%)	28 (45.2%)	12 (19.4%)
Colorectal cancer	42	17 (40.5%)	9 (21.4%)	0 (0%)
Liver cancer	20	8 (40.0%)	5 (25.0%)	1 (5.0%)
Pancreatic cancer	18	8 (44.4%)	3 (16.7%)	1 (5.6%)
Bladder cancer	34	33 (97.1%)	23 (67.6%)	11 (34.4%)
Testicular cancer	13	10 (76.9%)	7 (53.8%)	4 (30.8%)
AML	56	18 (32.1%)	6 (10.7%)	1 (1.8%)
Osteosarcoma	25	13 (52.0%)	6 (24.0%)	2 (8.0%)
Soft tissue tumor	63	51 (81.0%)	35 (55.6%)	16 (25.4%)

Abbreviations: NSCLC, non-small cell lung cancer; SCLC, small cell lung cancer; AML, acute myeloid leukemia.

*We compared the signal intensity of *MCM7 *between tumour tissues and corresponding non-neoplastic tissues derived from the same patient.

### MCM7 is required for cancer cell proliferation

**Figure 3:**
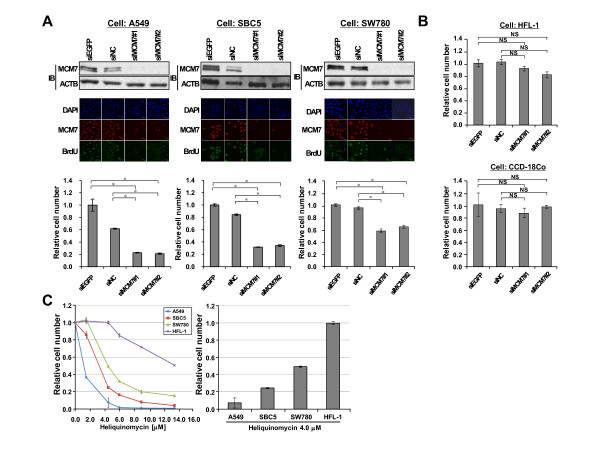
**Involvement of MCM7 in the proliferation of lung and bladder cancer cells**. (A) Knockdown effects of siRNAs targeting MCM7 on cancer cells. Top, western blot analysis of MCM7 in A549, SBC5 and SW780 cells after treatment with two MCM7 siRNAs (siMCM7#1 and siMCM7#2) and two control siRNAs (siEGFP and siNC). ACTB was used as an internal control. Middle, immunocytochemical analysis of MCM7 in A549 and SBC5 cells after treatment with siRNAs. The nucleus was stained with DAPI, and the incorporation of 5-bromo-2'-deoxyuridine (BrdU) into replicating DNA was used to label proliferating cells. Bottom, effects of MCM7 knockdown on the viability of lung and bladder cancer cell lines (A549, SBC5 and SW780). The viability of cancer cells was measured by Cell Counting Kit-8, and statistical analysis was done using Student's t-test. *, *P *< 0.05. (B) Effects of MCM7 siRNAs on the growth of HFL-1 cells and CCD-18Co cells. The viability of the cells was measured by Cell Counting Kit-8, and statistical analysis was done using Student's t-test. NS, not significant. (C) Effects of heliquinomycin on the viability of normal and cancer cells. Left, the cell vitality assay was performed using Cell Counting Kit-8 after treatment with heliquinomycin for 72 h. Results are the mean ± SD of three independent experiments. Right, the cell viability of A549, SBC5, SW780 and HFL-1 72 h after treatment with 4.0 μM heloquinomycin.

In order to examine whether elevated expression of MCM7 plays a critical role in the proliferation of cancer cells, we prepared siRNA oligonucleotide duplexes to specifically suppress the expression of *MCM7 *(siMCM7#1, #2), and transfected each of them into cancer cells. Firstly, we examined the expression profile of MCM7 in various types of normal and cancer cell lines. Quantitative real-time PCR and western blot analyses showed that MCM*7 *expression levels in cancer cell lines were significantly higher than those in normal human cell lines at the RNA and protein levels (Additional file 7). Knockdown of MCM7 in A549, SBC5 and SW780 cells was confirmed by western blot and immunocytochemical analyses as shown in Figure 3A. Immunocytochemical analysis showed a decreased proportion of cells incorporating BrdU after treatment with MCM7 siRNAs and this abrogation of cancer cell growth was confirmed by the cell viability assay using cell counting kit 8 (Figure 3A). After confirming that the normal HFL-1 and CCD-18Co cell lines show almost the same growth rate as the cancer cell lines (Additional file 8), we examined effects of these siRNAs on the normal cells (Figure 3B). The cell viability analysis revealed that MCM7 siRNAs had only slight effects on these normal cell lines relative to cancer cell lines, implying that the marked suppression of cancer cell growth by treatment with these specific siRNAs was not dependent on off-target effects. In addition, we demonstrated the effects of heliquinomycin, an inhibitor of MCM4/6/7-dependent DNA helicase activity [34], on the growth of cancer cells. Importantly, it effectively suppressed the growth of cancer cells compared with HFL-1 cells and this effect was in a dose dependent manner (Figure 3C). In particular, after treatment with 4.0 μM of heliquinomycin, no growth-suppressive effects were observed in HFL-1 cells even though significant growth suppression was observed in cancer cells. These data indicate that MCM7 plays an important role in the proliferation of cancer cells, and inactivation of this gene should be a promising therapeutic target in various types of cancer.

## Discussion

Six of the MCM proteins, MCM2-MCM7, form complexes that participate in initiation and elongation steps of DNA replication [35]. They share a conserved 200-amino acid nucleotide-binding region and form different subcomplexes (dimers, trimers and a hexamer) [36]. MCM4-MCM6-MCM7 trimers and hexamers (MCM2-MCM7) have ATPase and DNA helicase activities *in vitro *[35]. MCM proteins are associated with chromatin in late telophase and at the beginning of the G_1 _phase of the cell cycle [37]. During S phase, MCM proteins are released from origins of replication after initiation of DNA replication and subsequently move with replication folks where they are thought to function as a DNA helicase. Mechanisms that assure the replication of DNA only once per cycle involve the release of MCM proteins from chromatin after firing of the origins of replication and prevent the reloading of MCM proteins on chromatin until telophase. This evidence indicates that MCM proteins are one of the essential regulators in DNA replication, and indeed, it has already been reported that dysregulation of some MCM proteins is apparent in human diseases [38-43].

Mukherjee *et al *recently showed that Rb protein can bind the MCM complex during late G_1 _via a direct interaction with Mcm7, and TGF-β1 blocks their dissociation at G_1_/S [44]. Overexpression of Mcm7 can specifically and effectively abrogate the ability of TGF-β1 to acutely block cells in late G_1_. Since the Rb-binding C-terminal domain of Mcm7 is sufficient for this, Rb and Mcm7 can form complexes in late G_1 _that are targeted by TGF-β1 signals, and perturbation of the Rb-Mcm7 interaction by loss of Rb or overexpression of Mcm7 leads to disruption of TGF-β1's ability to block late-G_1 _transit into S phase [44]. These data imply that one reason for the involvement of MCM7 in human cancer we found may be due to its strong ability to promote cell cycle progression. Likewise, MCM2, a member of the family of MCM proteins, was reported as a promising marker for premalignant lesions of the lung and an independent predictor of survival in patients with NSCLC [39,45]. Together with our findings, deregulation of the MCM family protein appears to be closely linked to human carcinogenesis.

Cancer-related death is on the rise in most countries, and it is a serious public health problem. It is true that novel molecular-targeting agents such as cetuximab and bevacizumab have been developed and proven to be efficacious, but their adverse effects and limited application for some patients, engender a drive towards novel molecular-targeting agents [46-48]. Recent tumor genomics data are now assisting in the development of more rationally selected drugs that target proteins expressed exclusively or at particularly high levels in tumor compared with essential normal adult cells. It is hoped that the specific pharmaceutical targeting of such proteins will result in a new generation of highly active drugs that are associated with minimal collateral host toxicity [49]. In this study, we demonstrated that in various cancer tissues, expression levels of MCM7 were significantly high at both mRNA and protein levels whereas those in various normal tissues were generally low. Although knockdown of MCM7 expression by specific siRNAs notably suppressed the growth of cancer cells, significant growth suppression was hardly observed in the normal HFL-1 and CCD-18Co cells. Furthermore, an optimal dose of heliquinomycin, a specific inhibitor of MCM4/6/7-dependent DNA helicase activity, more effectively inhibited the growth of cancer cell lines than that of HFL-1 cell line. These data imply optimized inhibition of MCM7 activity may be a new strategy for cancer therapy. Importantly, we found that the growth rate between human normal and cancer cell lines in culture (Additional file 8) was not very different, even though expression levels of MCM7 in cancer cell lines were significantly higher than those in normal cell lines, indicating that overexpressed MCM7 in cancer cells may relate to some tumor-specific functions, including growth regulation. This kind of evidence also reinforces the importance of MCM7 as a target for cancer therapy.

On the contrary, it is known that MCM7 positive cells are also located in proliferative zone, especially in gastrointestinal tract, as well as germinal center cells in lymph nodes. In this case, we quantitatively compared MCM7 expression levels in normal gastrointestinal tract tissues and lung cancer tissues, and the result showed that expression levels of *MCM7 *in lung cancer tissues are significantly higher than those in gastrointestinal tract normal tissues (Additional file 9). The data imply that expression levels of MCM7 in lung cancer tissues appear to be significantly higher than those in some MCM7-expressing normal tissues like gastrointestinal tissues. Meanwhile, we quantitatively analyzed MCM7 expression levels in 78 normal tissues and found that MCM7 expression was high in some normal tissues such as lymphoblasts compared with other normal tissues (Additional file 10). Therefore, we should take this kind of evidence into consideration when developing anti-cancer therapy targeting MCM7.

In addition, we clarified the tumor-related arginine methyltransferase PRMT6 could interact with MCM7 in the present study. Since both of these genes were overexpressed in tumor tissues, it is possible that these gene products may cooperatively contribute to human carcinogenesis. Although we conducted *in vitro *methyltransferase to examine the possibility that MCM7 can serve as a substrate of PRMT6, we failed to secure the critical evidence (Additional file 11). With regard to this matter, we have also been examining the possibility that other MCM family proteins can interact with PRMT6 and serve as substrates. Further functional analyses may make clear the cooperation of PRMT6 in the MCM complex in human cancer.

## Conclusions

Our new findings indicate that MCM7 may be an ideal target for molecular targeted therapy of cancer without severe side effects. As anti-cancer drugs targeting DNA helicases are now in development [50], further validation of present results may affirm the importance of this protein as a promising target for anti-cancer therapy. Furthermore, among all types of cancer, lung cancer is the leading cause of death from cancer in the United States and Japan, and the median survival of advanced NSCLC patients treated with standard chemotherapy still remains as short as about 8 months [51,52]. MCM7 could therefore be a good indicator enabling us to predict prognosis of NSCLC patients and to conduct a more intensive follow-up according to MCM7 expression status of resected specimens.

## Abbreviations

NSCLC: non-small cell lung cancer; SCLC: small cell lung cancer.

## Authors' contributions

GT, YN and RH designed this study and performed all experiments with the help of KM, H-SC, MY, MT, SH and KM. ET and DEN kindly provided patient samples and gave good advice. YD, MC, HIF, BAJP, YM and YN critically read the manuscript and gave good advice. GT and RH wrote this manuscript. All authors read and approved final manuscript.

## Conflict of interests

The authors declare that they have no competing interests.

## Supplementary Material

Additional file  1 **Primer sequences for quantitative RT-PCR**. Specific primer sequence for *GAPDH *(housekeeping gene) and *MCM7*, respectively. Click here for file

Additional file  2 **siRNA sequences**. Sequences of siEGFP, siNC (Negative Control) and siMCM7, respectively. Click here for file

Additional file  3 **PRMT6 associates with MCM7**. (A) FLAG-mock or FLAG-PRMT6 expression vectors were co-transfected with HA-Mock or HA-MCM7 expression vectors into 293T cells. After 48 h, cells were immunoprecipitated with anti-FLAG M2 agarose (SIGMA) or -HA agarose (SIGMA), and immunoprecipitants were immunoblotted with anti-FLAG and -HA antibodies, respectively. (B) FLAG-PRMT6 could interact with endogenous MCM7 proteins. FLAG-Mock or FLAG-PRMT6 expression vectors were transfected into 293T cells. After 48 h, cells were immunoprecipitated with anti-FLAG M2 agarose, and immunoprecipitants were immunoblotted with anti-MCM7 and -FLAG antibodies, respectively. (C) PRMT6 and MCM7 were co-localized in the nucleus. HeLa cells were stained with anti-FLAG antibody (Alexa Fluor^® ^488 [green]), anti-HA antibody (Alexa Fluor^® ^594 [red]) and 4',6'-diamidine-2'-phenylindole dihydrochloride (DAPI [blue]). Click here for file

Additional file  4 **Clinicopathological characteristics of bladder tissues on the tissue microarray**. Clinicopathological information of bladder tumor tissues and MCM7 expression levels at the protein level. Click here for file

Additional file  5 **Immunohistochemical analysis of MCM7 in liver cancer tissues**. Original magnification, ×200. Click here for file

Additional file  6 **Clinicopathological characteristics of liver tissues on the tissue microarray**. Clinicopathological information of liver tumor tissues and MCM7 expression levels at the protein level. Click here for file

Additional file  7 **Expression levels of MCM7 in various types of cancer cell lines**. (A) mRNA expression levels of *MCM7 *in 5 normal human cell lines, 15 lung cancer cell lines, 3 bladder-cancer cell lines, 3 liver cancer cell lines, and 3 colorectal cancer cell lines examined by quantitative real-time PCR. (B) Protein expression levels of MCM7 in HFL-1, A549, SBC5 and SW780 cell lines examined by western blot. ACTB serves as a loading control. Click here for file

Additional file  8 **The growth rate of various types of cell lines**. Cell growth was calculated using Cell Counting Kit-8 and cell number shows the relative value compared to that at 0 h (cell number at 0 h = 1). The experiment was performed during the logarithmic growth phase. Click here for file

Additional file  9 **Expression levels of MCM7 in gastrointestinal tract (GIT) normal tissues (colon, esophagus, rectum and stomach) and lung cancer tissues**. (A) mRNA expression levels of *MCM7 *in GIT normal tissues and lung cancer tissues examined by quantitative real-time PCR. (B) Comparison of *MCM7 *expression between GIT normal tissues and lung cancer tissues based on the real-time PCR result shown in (A). Mann-Whitney's *U-*test was used for statistical analysis, and data are shown by box-whisker plot (median 50% boxed). Click here for file

Additional file  10 **Expression levels of *MCM7 *in 78 normal tissues**. The data were derived from BioGPS http://biogps.gnf.org/#goto=welcome. *GAPDH *expression is shown as a control for signal intensity. Click here for file

Additional file  11 **MCM7 was not methylated by PRMT6**. For the *in vitro *methyltransferase assay, recombinant MCM7 proteins were incubated with active PRMT6 using 2 μCi S-adenosyl-L-[methyl-^3^H] methionine (SAM; Amersham Biosciences) as the methyl donor in a mixture of 10 μl of methylase activity buffer (50 mM Tris-HCl at pH8.5, 10 mM DTT and 10 mM MgCl_2_), for 1 h at 30°C. Proteins were resolved on a 5-20% SDS-PAGE gel (Ready Gel; Bio-Rad, Hercules, CA, USA) and visualized by fluorographyand ponceau S staining. Click here for file

Additional file  12 **Characteristics of various normal tissues**. Clinical information of normal organs. Click here for file
