# Supplementary material for: Minichromosome Maintenance Protein 7 is a potential therapeutic target in human cancer and a novel prognostic marker of non-small cell lung cancer
Source: Mol Cancer. 2011 May 28;10:65. doi: 10.1186/1476-4598-10-65 (PMC3125391; doi:10.1186/1476-4598-10-65)
Supplement: Additional file 1 — Primer sequences for quantitative RT-PCR. Specific primer sequence for GAPDH (housekeeping gene) and MCM7, respectively. [file 1476-4598-10-65-S1.PDF]

| Gene name | Primer sequence |
|---|---|
| *GAPDH* (housekeeping gene) - f | 5' GCAAATTCCATGGCACCGTC 3’ |
| *GAPDH* (housekeeping gene) - r | 5' TCGCCCCACTTGATTTTGG 3’ |
| *MCM7* - f | 5' CAGAACCAGTACCCTGCTGAAC 3' |
| *MCM7* - r | 5' ATTCCACGCACAGTTACCAAC 3' |
