# Supplementary material for: Minichromosome Maintenance Protein 7 is a potential therapeutic target in human cancer and a novel prognostic marker of non-small cell lung cancer
Source: Mol Cancer. 2011 May 28;10:65. doi: 10.1186/1476-4598-10-65 (PMC3125391; doi:10.1186/1476-4598-10-65)
Supplement: Additional file 2 — siRNA sequences. Sequences of siEGFP, siNC (Negative Control) and siMCM7, respectively. [file 1476-4598-10-65-S2.PDF]

siRNA name		Sequence
siEGFP		Sense: 5’ GCAGCACGACUUCUUCAAGTT 3’<br>Antisense: 5’ CUUGAAGAAGUCGUGCUGCTT 3’
siNegative control (Cocktail)	Target#1	Sense: 5’ AUCCGCGCGAUAGUACGUA 3’<br>Antisense: 5’ UACGUACUAUCGCGCGGAU 3’
	Target#2	Sense: 5’ UUACGCGUAGCGUAAUACG 3’<br>Antisense: 5’ CGUAUUACGCUACGCGUAA 3’
	Target#3	Sense: 5’ UAUUCGCGCGUAUAGCGGU 3’<br>Antisense: 5’ ACCGCUAUACGCGCGAAUA 3’
siMCM7 #1		Sense: 5’ GGCUAAUGGAGAUGUCAA 3’<br>Antisense: 5’ UUUGACAUCUCCAUUAGCCTT 3’
siMCM7 #2		Sense: 5’ GAAAGAAGAUGUGAAUGA 3’<br>Antisense: 5’ UUCAUUCACAUCUUCUUUCTT 3’
