# Supplementary figures and images for: Minichromosome Maintenance Protein 7 is a potential therapeutic target in human cancer and a novel prognostic marker of non-small cell lung cancer
Source: Mol Cancer. 2011 May 28;10:65. doi: 10.1186/1476-4598-10-65 (PMC3125391; doi:10.1186/1476-4598-10-65)

**A**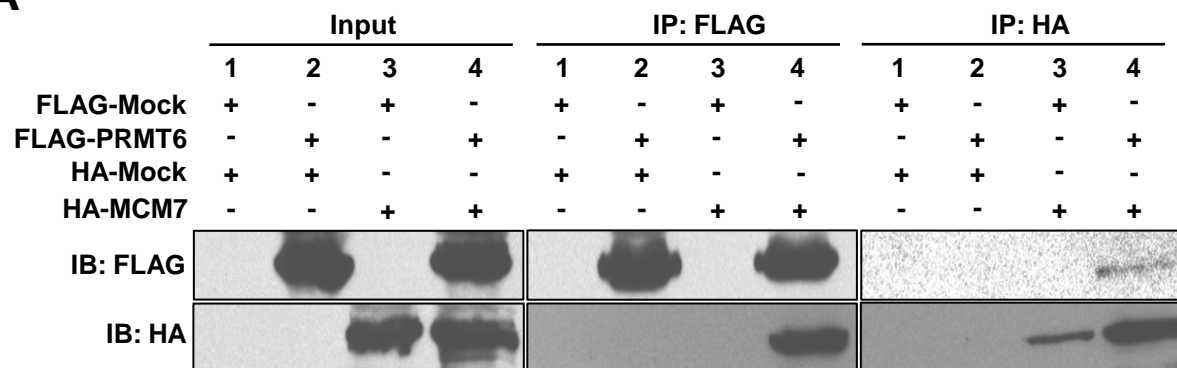**B**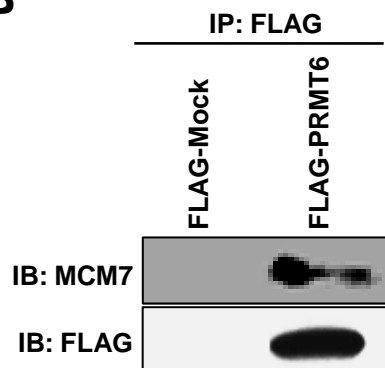**C**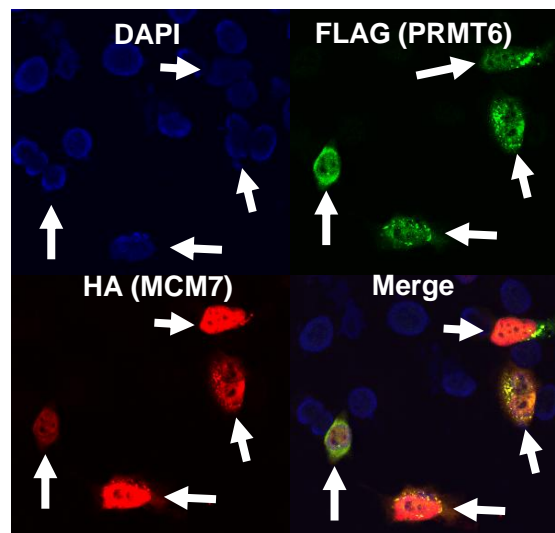

Supplement: Additional file 3 — PRMT6 associates with MCM7. (A) FLAG-mock or FLAG-PRMT6 expression vectors were co-transfected with HA-Mock or HA-MCM7 expression vectors into 293T cells. After 48 h, cells were immunoprecipitated with anti-FLAG M2 agarose (SIGMA) or -HA agarose (SIGMA), and immunoprecipitants were immunoblotted with anti-FLAG and -HA antibodies, respectively. (B) FLAG-PRMT6 could interact with endogenous MCM7 proteins. FLAG-Mock or FLAG-PRMT6 expression vectors were transfected into 293T cells. After 48 h, cells were immunoprecipitated with anti-FLAG M2 agarose, and immunoprecipitants were immunoblotted with anti-MCM7 and -FLAG antibodies, respectively. (C) PRMT6 and MCM7 were co-localized in the nucleus. HeLa cells were stained with anti-FLAG antibody (Alexa Fluor® 488 [green]), anti-HA antibody (Alexa Fluor® 594 [red]) and 4',6'-diamidine-2'-phenylindole dihydrochloride (DAPI [blue]). [file 1476-4598-10-65-S3.PDF]

**A**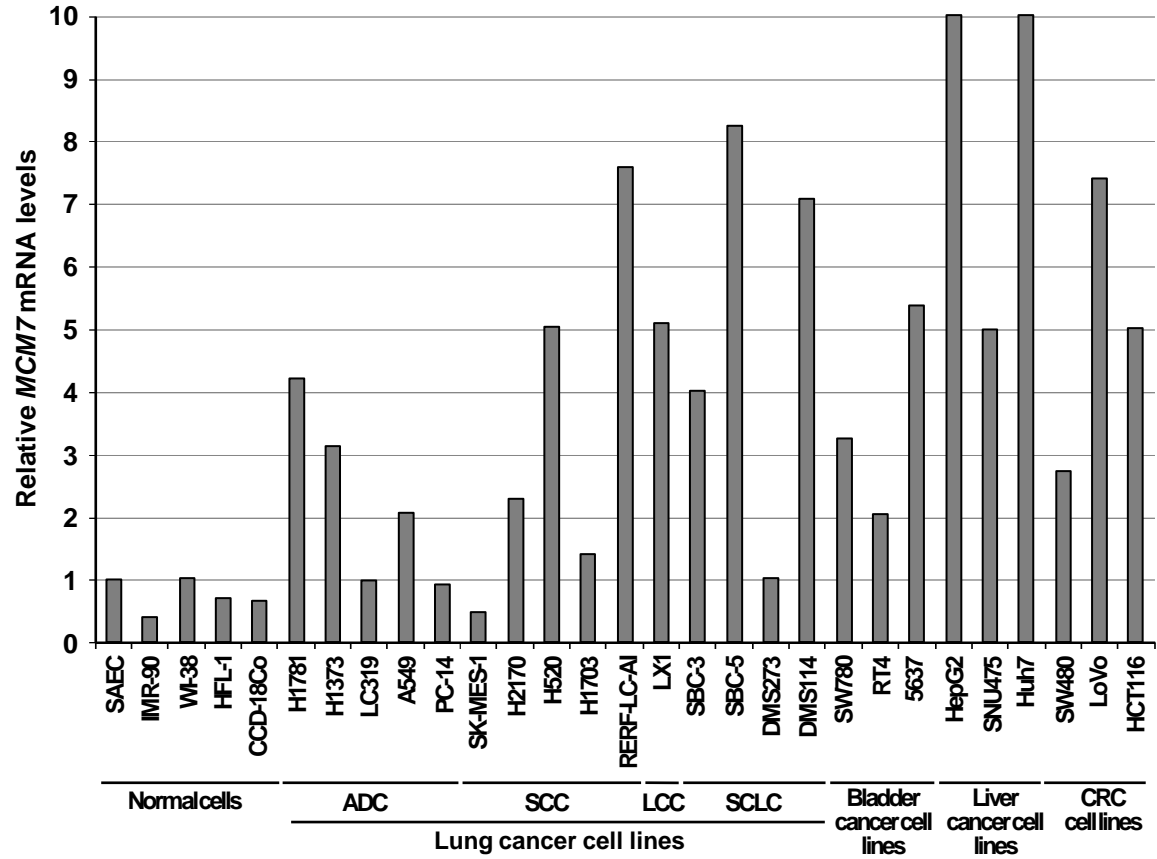**B**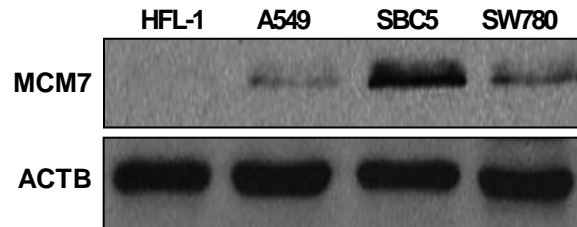

Supplement: Additional file 7 — Expression levels of MCM7 in various types of cancer cell lines. (A) mRNA expression levels of MCM7 in 5 normal human cell lines, 15 lung cancer cell lines, 3 bladder-cancer cell lines, 3 liver cancer cell lines, and 3 colorectal cancer cell lines examined by quantitative real-time PCR. (B) Protein expression levels of MCM7 in HFL-1, A549, SBC5 and SW780 cell lines examined by western blot. ACTB serves as a loading control. [file 1476-4598-10-65-S7.PDF]

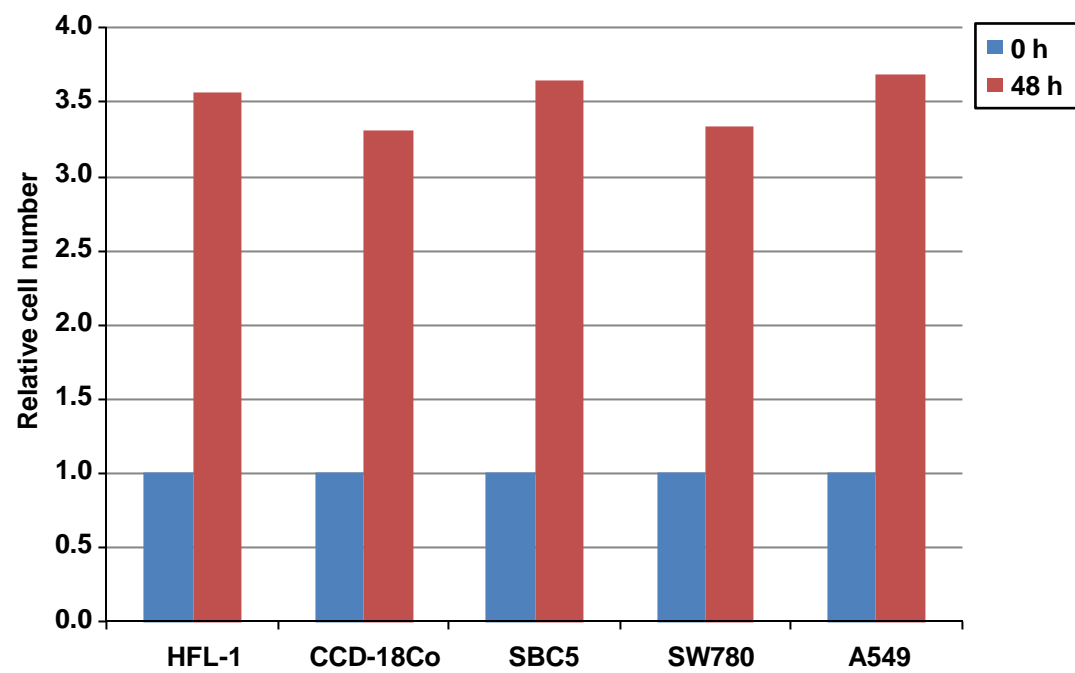

Supplement: Additional file 8 — The growth rate of various types of cell lines. Cell growth was calculated using Cell Counting Kit-8 and cell number shows the relative value compared to that at 0 h (cell number at 0 h = 1). The experiment was performed during the logarithmic growth phase. [file 1476-4598-10-65-S8.PDF]

**A**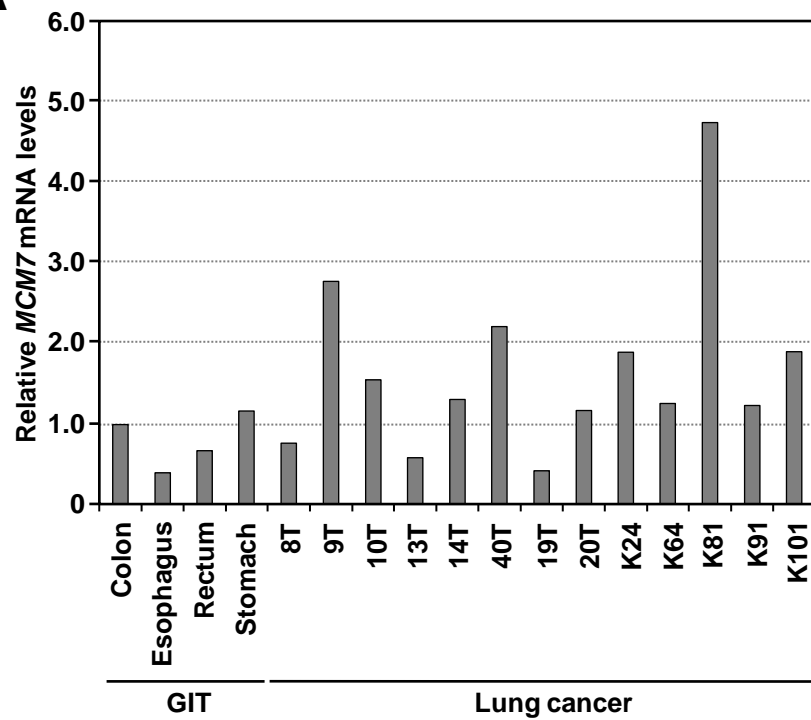**B**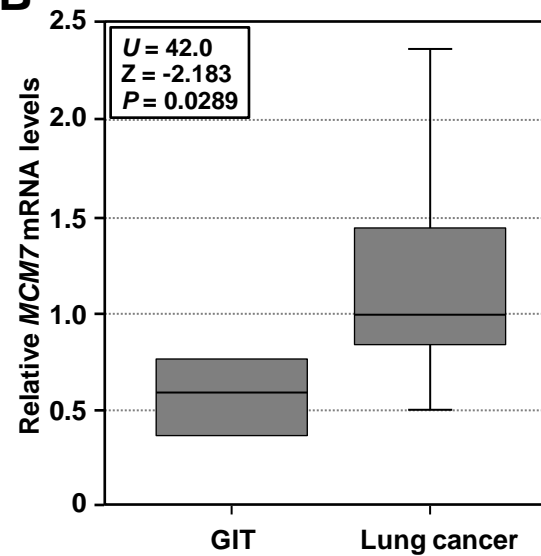

Supplement: Additional file 9 — Expression levels of MCM7 in gastrointestinal tract (GIT) normal tissues (colon, esophagus, rectum and stomach) and lung cancer tissues. (A) mRNA expression levels of MCM7 in GIT normal tissues and lung cancer tissues examined by quantitative real-time PCR. (B) Comparison of MCM7 expression between GIT normal tissues and lung cancer tissues based on the real-time PCR result shown in (A). Mann-Whitney's U-test was used for statistical analysis, and data are shown by box-whisker plot (median 50% boxed). [file 1476-4598-10-65-S9.PDF]

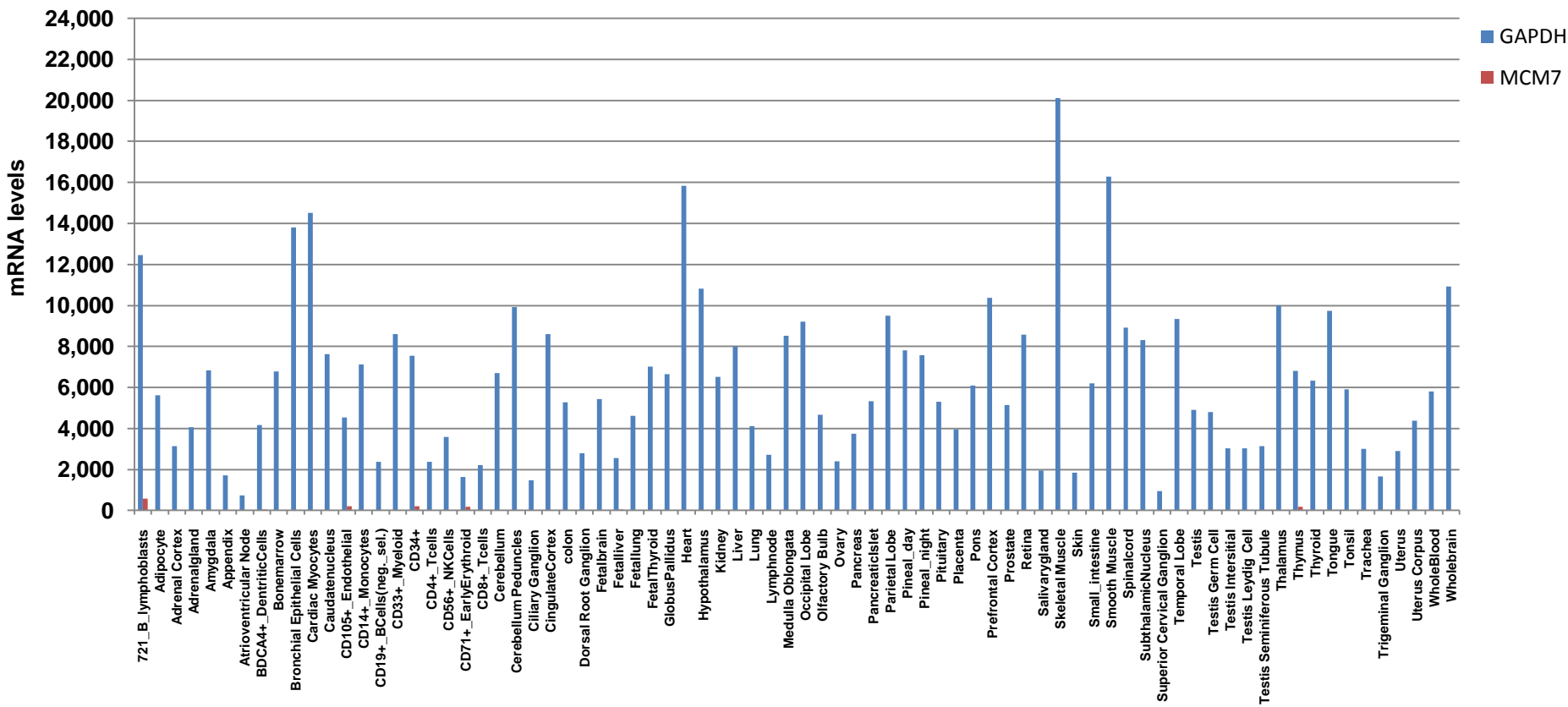

Supplement: Additional file 10 — Expression levels of MCM7 in 78 normal tissues. The data were derived from BioGPS http://biogps.gnf.org/#goto=welcome. GAPDH expression is shown as a control for signal intensity. [file 1476-4598-10-65-S10.PDF]

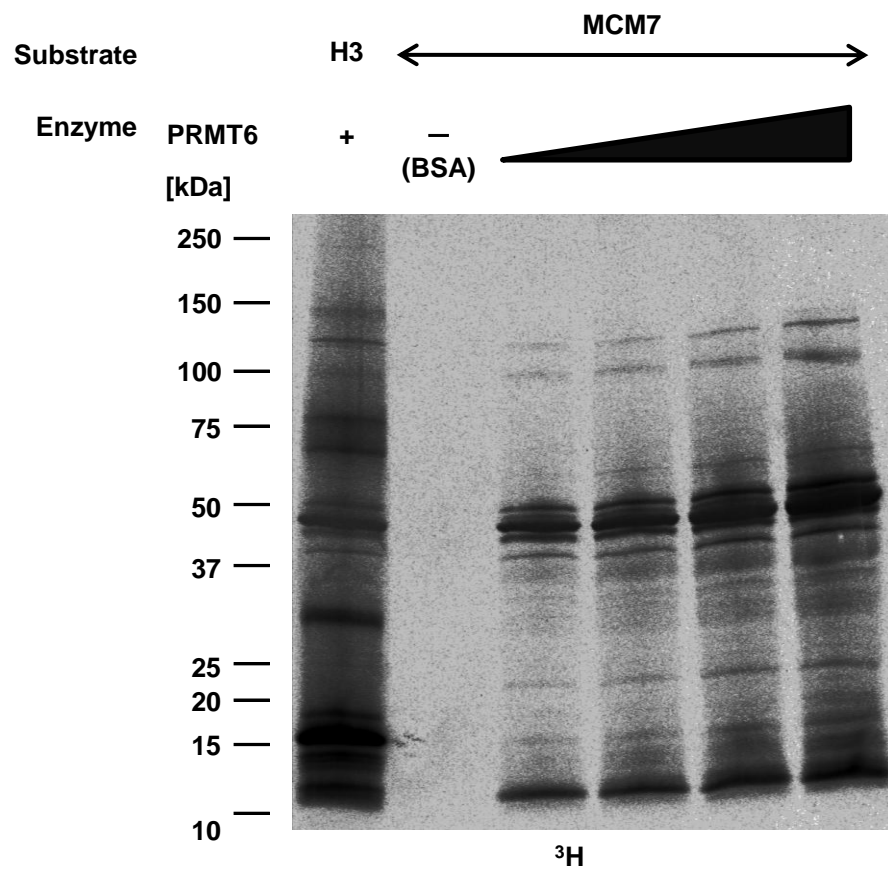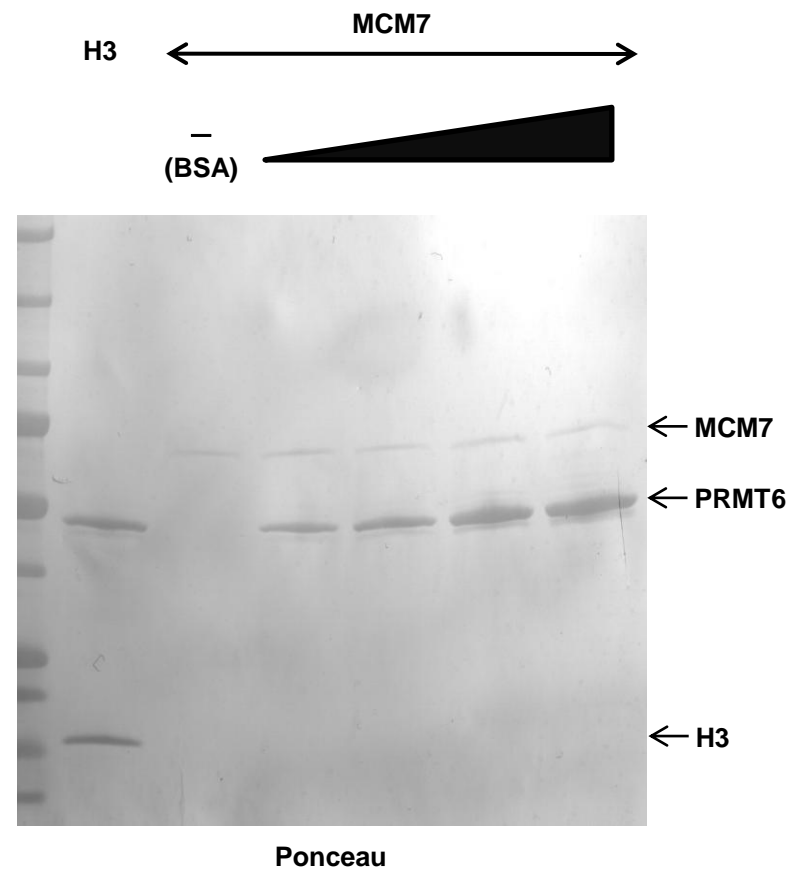

Supplement: Additional file 11 — MCM7 was not methylated by PRMT6. For the in vitro methyltransferase assay, recombinant MCM7 proteins were incubated with active PRMT6 using 2 μCi S-adenosyl-L-[methyl-3H] methionine (SAM; Amersham Biosciences) as the methyl donor in a mixture of 10 μl of methylase activity buffer (50 mM Tris-HCl at pH8.5, 10 mM DTT and 10 mM MgCl2), for 1 h at 30°C. Proteins were resolved on a 5-20% SDS-PAGE gel (Ready Gel; Bio-Rad, Hercules, CA, USA) and visualized by fluorographyand ponceau S staining. [file 1476-4598-10-65-S11.PDF]
