# Supplementary material for: Minichromosome Maintenance Protein 7 is a potential therapeutic target in human cancer and a novel prognostic marker of non-small cell lung cancer
Source: Mol Cancer. 2011 May 28;10:65. doi: 10.1186/1476-4598-10-65 (PMC3125391; doi:10.1186/1476-4598-10-65)
Supplement: Additional file 4 — Clinicopathological characteristics of bladder tissues on the tissue microarray. Clinicopathological information of bladder tumor tissues and MCM7 expression levels at the protein level. [file 1476-4598-10-65-S4.PDF]

Sample Number	Age	Gender	Histology	Grade	Stage (TNM)	MCM7 expression
1	71	M	Normal	-	-	-
2	59	M	Normal	-	-	-
3	65	M	Chronic cystitis	-	-	-
4	51	F	Chronic cystitis	-	-	-
5	71	M	Squamous cell carcinoma	I	T1N0M0	+
6	60	M	Squamous cell carcinoma	I	T2N0M0	-
7	76	M	Adenocarcinoma	II	T2N0M0	+
8	50	M	Adenocarcinoma	II	T2N0M0	+
9	68	M	Adenocarcinoma	III	T2N0M0	-
10	74	F	Adenocarcinoma	III	T2N0M0	+
11	27	M	Transitional cell carcinoma	I	TisN0M0	+
12	50	M	Transitional cell carcinoma	I	T1N0M0	+
13	49	F	Transitional cell carcinoma	I	T1N0M0	+
14	67	M	Transitional cell carcinoma	I	T1N0M0	+
15	51	F	Transitional cell carcinoma	I	T1N0M0	+
16	57	M	Transitional cell carcinoma	I	T1N0M0	+
17	47	M	Transitional cell carcinoma	II	T2N0M0	+
18	54	M	Transitional cell carcinoma	II	T2N0M0	+
19	45	M	Transitional cell carcinoma	II	T1N0M0	-
20	74	M	Transitional cell carcinoma	II	T2N0M0	+
21	51	M	Transitional cell carcinoma	II	T1N0M0	+
22	80	M	Transitional cell carcinoma	II	T2N0M0	-
23	53	F	Transitional cell carcinoma	II	T1N0M0	-
24	37	M	Transitional cell carcinoma	II	T2N0M0	+
25	55	M	Transitional cell carcinoma	II	T4N2MX	+
26	52	M	Transitional cell carcinoma	II	T1N0M0	-
27	78	M	Transitional cell carcinoma	III	T1N0M0	-
28	64	M	Transitional cell carcinoma	III	T3N2M1	+
29	70	M	Transitional cell carcinoma	III	T2N0M0	+
30	61	M	Transitional cell carcinoma	III	T2N0M0	+
31	61	M	Transitional cell carcinoma	III	T1N0M0	+
32	39	F	Transitional cell carcinoma	III	T2N0M0	+
33	30	M	Sarcoma	-	T2N0M0	-

*All tissue samples were purchased from BioChain

(-) negative expression
(+) positive expression
