# Supplementary material for: Minichromosome Maintenance Protein 7 is a potential therapeutic target in human cancer and a novel prognostic marker of non-small cell lung cancer
Source: Mol Cancer. 2011 May 28;10:65. doi: 10.1186/1476-4598-10-65 (PMC3125391; doi:10.1186/1476-4598-10-65)
Supplement: Additional file 5 — Immunohistochemical analysis of MCM7 in liver cancer tissues. Original magnification, ×200. [file 1476-4598-10-65-S5.PDF]

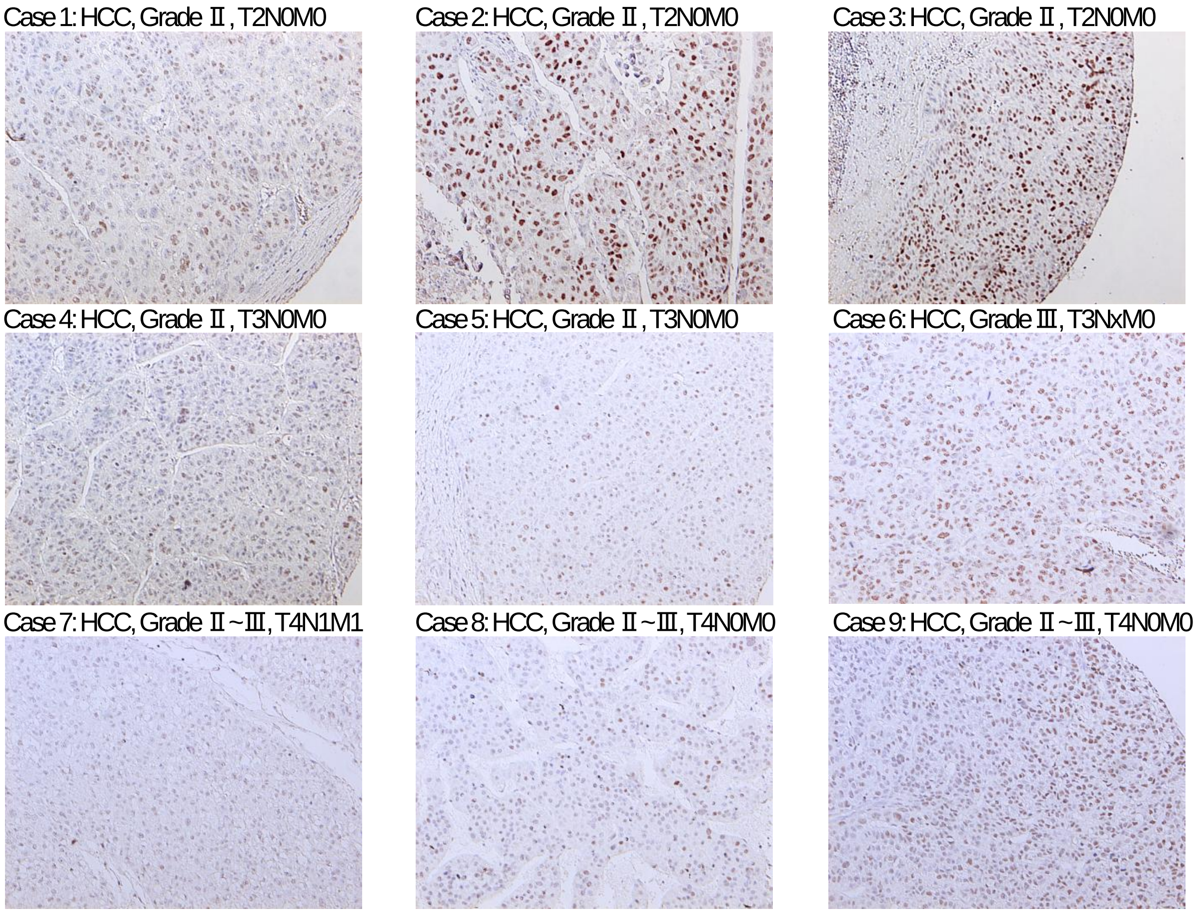
Case 1: HCC, Grade Ⅱ, T2N0M0
Case 2: HCC, Grade Ⅱ, T2N0M0
Case 3: HCC, Grade Ⅱ, T2N0M0
Case 4: HCC, Grade Ⅱ, T3N0M0
Case 5: HCC, Grade Ⅱ, T3N0M0
Case 6: HCC, Grade Ⅲ, T3NxM0
Case 7: HCC, Grade Ⅱ~Ⅲ, T4N1M1
Case 8: HCC, Grade Ⅱ~Ⅲ, T4N0M0
Case 9: HCC, Grade Ⅱ~Ⅲ, T4N0M0
