# Supplementary material for: Minichromosome Maintenance Protein 7 is a potential therapeutic target in human cancer and a novel prognostic marker of non-small cell lung cancer
Source: Mol Cancer. 2011 May 28;10:65. doi: 10.1186/1476-4598-10-65 (PMC3125391; doi:10.1186/1476-4598-10-65)
Supplement: Additional file 6 — Clinicopathological characteristics of liver tissues on the tissue microarray. Clinicopathological information of liver tumor tissues and MCM7 expression levels at the protein level. [file 1476-4598-10-65-S6.PDF]

Sample Number	Age	Gender	Histology	Grade	Stage (TNM)	MCM7 expression
1	48	M	Hepatocellular carcinoma	II~III	T3NxM0	-
2	72	F	Bile duct carcinoma	I~II	T3N0M0	-
3	45	F	Bile duct carcinoma	II	T3N1M1	-
4	35	F	Hepatocellular carcinoma	III	T3N0M0	-
5	57	M	Hepatocellular carcinoma	III	T3N0M0	-
6	47	M	Hepatocellular carcinoma	II~III	T3N0M0	-
7	31	F	Hepatocellular carcinoma	II~III	T3N0M0	-
8	49	M	Hepatocellular carcinoma	II~III	T3N0M0	-
9	54	M	Hepatocellular carcinoma	III	T4N1M0	-
10	33	M	Hepatocellular carcinoma	II~III	T3N0M0	-
11	40	M	Hepatocellular carcinoma	I~II	T3N0M0	-
12	55	F	Hepatocellular carcinoma	II	T3N0M0	-
13	50	M	Hepatocellular carcinoma	II~III	T3N0M0	-
14	47	M	Hepatocellular carcinoma	II~III	T3N0M0	-
15	79	M	Hepatocellular carcinoma	II~III	T3N0M0	-
16	44	M	Hepatocellular carcinoma	II~III	T3N0M0	+
17	45	M	Hepatocellular carcinoma	III	T3N0M0	+
18	48	M	Clear cell carcinoma	III	T3N0M0	+
19	59	M	Hepatocellular carcinoma	II~III	T4N0M0	+
20	35	F	Hepatocellular carcinoma	II	T3N0M0	+
21	42	M	Hepatocellular carcinoma	II	T3N0M0	+
22	57	F	Hepatocellular carcinoma	II~III	T4N1M1	+
23	61	M	Hepatocellular carcinoma	II~III	T4N0M0	+
24	44	M	Hepatocellular carcinoma	II	T3N0M0	+
25	37	F	Hepatocellular carcinoma	II	T4N0M0	+
26	35	M	Hepatocellular carcinoma	II	T3N0M0	-
27	35	F	Hepatocellular carcinoma	II	T3N0M0	-
28	43	F	Hepatocellular carcinoma	II	T2N0M0	-
29	38	M	Hepatocellular carcinoma	II	T2N0M0	-
30	30	M	Hepatocellular carcinoma	II	T2N0M0	+
31	36	M	Hepatocellular carcinoma	I	T3N0M0	+
32	39	M	Hepatocellular carcinoma	II	T2N0M0	+
33	77	M	Hepatocellular carcinoma	II	T2N0M0	+
34	29	M	Hepatocellular carcinoma	I	T2N0M0	+
35	77	M	Hepatocellular carcinoma	II~III	T2N0M0	+

(-) negative expression
(+) positive expression

Sample Number	Age	Gender	Histology	Grade	Stage (TNM)	MCM7 expression
36	60	F	Leiomyosarcoma?			-
37	41	M	Hepatocellular carcinoma	II~III	T2N0M0	+
38	39	M	Clear cell carcinoma	II	T2N0M0	-
39	38	F	Hepatocellular carcinoma	II~III	T2N0M0	+
40	69	M	Hepatocellular carcinoma	II	T3N0M0	+
41	38	M	Hepatocellular carcinoma	II	T3N0M0	-
42	76	M	Hepatocellular carcinoma	II	T3N0M0	-
43	38	M	Hepatocellular carcinoma	I~II	T3N0M0	-
44	30	M	Bile duct carcinoma	II	T2N0M0	-
45	1.5	M	Hepatoblastoma			-
46	52	M	Hepatocellular carcinoma	I~II	T3N0M0	-
47	56	M	Hepatocellular carcinoma	II	T4N0M0	-
48	56	M	Hepatocellular carcinoma	II	T4N1M0	-
49	49	M	Hepatocellular carcinoma	II	T4N0M0	-
50	57	M	Hepatocellular carcinoma	II~III	T4N1M0	-
51	40	F	Hepatocellular carcinoma	II	T2N0M0	-
52	61	M	Hepatocellular carcinoma	II~III	T4N1M1	-
53	73	F	Clear cell carcinoma	II	T3N0M0	-
54	41	M	Hepatocellular carcinoma	II	T2N0M0	+
55	58	F	Hepatocellular carcinoma	III	T2N0M0	+
56	43	F	Hepatocellular carcinoma	II	T2N0M0	-
57	67	M	Hepatocellular carcinoma	II	T2N0M0	-
58	50	M	Hepatocellular carcinoma	I	T2N0M0	-
59	52	F	Hepatocellular carcinoma	II	T2N0M0	-
60	54	M	Hepatocellular carcinoma	II	T2N0M0	-
61	40	M	Hepatocellular carcinoma	II	T2N0M0	-
62	56	M	Hepatocellular carcinoma	II	T3N0M0	-
63	31	M	Hepatocellular carcinoma	II	T2N0M0	+
64	58	M	Hepatocellular carcinoma	II	T2N0M0	+
65	49	M	Hepatocellular carcinoma	II	T2N0M0	-
66	48	M	Hepatocellular carcinoma	I	T2N0M0	-
67	35	M	Hepatocellular carcinoma	III	T2N0M0	-
68	44	M	Hepatocellular carcinoma	II	T2N0M0	-
69	45	F	Fibrosarcoma?			+
70	53	M	Hepatocellular carcinoma	II	T3N0M0	+

*All tissue samples were purchased from BioChain

(-) negative expression
(+) positive expression
