# Supplementary material for: Minichromosome Maintenance Protein 7 is a potential therapeutic target in human cancer and a novel prognostic marker of non-small cell lung cancer
Source: Mol Cancer. 2011 May 28;10:65. doi: 10.1186/1476-4598-10-65 (PMC3125391; doi:10.1186/1476-4598-10-65)
Supplement: Additional file 12 — Characteristics of various normal tissues. Clinical information of normal organs. [file 1476-4598-10-65-S12.PDF]

**Patient ID**	**Age/Gender**	**Histology**	**Anatomic site**
Case 1	NA	Normal	Brain
Case 2	26/F	Normal	Heart
Case 3	59/M	Normal	Lung
Case 4	26/M	Normal	Liver
Case 5	NA	Normal	Pancreas
Case 6	NA	Normal	Stomach
Case 7	23/M	Normal	Testis
Case 8	44/M	Normal	Kidney
Case 9	59/M	Normal	Bladder

*All tissue samples were purchased from BioChain
Abbreviation: NA, not available
